# The cytokine signature in multiple sclerosis: a study during the SARS-CoV-2 pandemic

**DOI:** 10.3389/fimmu.2026.1775596

**Published:** 2026-03-16

**Authors:** Marco Puthenparampil, Annachiara Marin, Federica De Napoli, Alessandro Di Paola, Annamaria Valentina Mauceri, Susanna Ruggero, Francesca Rinaldi, Paola Perini, Antonella Viola, Barbara Molon, Paolo Gallo

**Affiliations:** 1 Department of Neurosciences – DNS, University of Padua, Padua, Italy; 2 Multiple Sclerosis Centre, Azienda Ospedaliera di Padova, Padua, Italy; 3 Immune-Mediated Nervous System Disease Study Group, Fondazione Istituto di Ricerca Pediatrica Città della Speranza, Padova, Italy; 4 Department of Biomedical Sciences, University of Padua, Padua, Italy; 5 Neuroradiology Unit, Azienda Ospedaliera di Padova, Padua, Italy

**Keywords:** cerebrospinal fluid, cytokines, multiple sclerosis, peripheral immune activation, SARS-CoV-2

## Abstract

**Background:**

Multiple sclerosis (MS) is a multifactorial autoimmune disorder resulting from the interplay of genetic susceptibility and environmental exposures. Viral infections, particularly Epstein–Barr virus (EBV), have been implicated in disease pathogenesis through mechanisms such as molecular mimicry. The SARS-CoV-2 pandemic provided a unique opportunity to explore whether large-scale viral exposure influenced early MS immunopathogenesis.

**Methods:**

In this cross-sectional study, we compared cytokine profiles in patients with first MS onset during 2020 (pandMS, *n* = 36) with age-, sex-, and disease duration-matched pre-pandemic MS cases (MS, *n* = 20) and a reference cohort of individuals with other non-inflammatory neurological diseases (ONIND, *n* = 20). Paired cerebrospinal fluid (CSF) and serum samples were analyzed for 45 cytokines, along with neurofilament light (NfL) chain, BAFF, and CXCL13. Intrathecal cytokine synthesis was estimated using CSF/serum quotients and indices. Magnetic resonance imaging (MRI) and clinical evaluations, including Expanded Disability Status Scale (EDSS), were conducted in a patient subset. Statistical analyses included correlation, logistic regression, and multivariate modelling.

**Results:**

CXCL13 and BAFF were elevated in both MS and pandMS, consistent with B-cell recruitment and survival. A second cytokine cluster (marked by CCL4) indicative of astrocyte–microglia activation increased exclusively in MS. In pandMS, peripheral IL-7, PDGF-BB, and CCL2 were selectively elevated. Notably, serum and CSF CCL2 correlated only in pandMS, and serum CCL2 associated with white matter lesion burden. Logistic regression distinguished pandMS from MS based on CXCL13 index, CCL4 index, and serum PDGF-BB (*r*² = 0.65). MRI lesion counts and clinical parameters did not differ between groups.

**Conclusions:**

While core MS-associated cytokines remain temporally stable, the pandemic period was associated with a shift toward peripheral immune activation at disease onset in pandMS. These findings likely reflect environmental and behavioral changes during the SARS-CoV-2 pandemic rather than direct viral effects. Our results highlight the dynamic interplay between central and peripheral immune mechanisms in early MS and reinforce the potential role of environmental exposures in modulating disease immunopathogenesis.

## Introduction

Multiple sclerosis (MS) has a complex and multifactorial etiology that involves the interplay of genetic susceptibility factors, i.e., genes involved in immune system regulation, and environmental factors, including vitamin D, smoking, obesity, and infectious agents (1–4). Among them, several viruses have been suspected to be involved in MS etiology [i.e., measles, papovaviruses, retroviruses, and Epstein–Barr virus (EBV)], but the exact mechanism that link anti-viral immunological responses and autoimmunity has remained elusive (5–7). Indeed, only for EBV has a clear CD4^+^ T-cell clone reactivity to both EBNA1 antigens and myelin-derived peptides been demonstrated, strongly supporting molecular mimicry (8). More recently, anti-EBNA1 Ig was found to recognize alpha-crystallin B (CRYAB) (9). These antibodies, produced by memory B cells, were increased in the supernatant of anti-EBNA1_380–641_ IgG, and positively correlated with anti-GlialCAM_262–416_ and anti-ANO_21-275_ (10). Taken together, these findings link anti-EBV immunological response to a self-antigen associated with MS.

In the last 4 years, a new virus faced humans and the immune system, the SARS-CoV-2 virus, belonging to the coronaviruses family that was used in the 1980s to induce the experimental autoimmune encephalomyelitis (EAE) murine model of MS in Lewis rats. In that model, the murine coronavirus JHM was found to induce the development of myelin basic protein (MBP)-reactive T cells that causes EAE following the infection (11), thus suggesting that, also in humans, coronavirus infection might induce pro-inflammatory changes in the immune system and even trigger autoimmune disorders.

The SARS-CoV-2 pandemic gave the possibility of exploring *in vivo* this hypothesis because an infective agent spread throughout the population in 2020, also affecting people who developed MS. To what extent the immunopathogenesis of MS could have been modified by SARS-CoV-2 contact was investigated in a cross-sectional study, which evaluated a wide range of cytokines in patients who developed MS before and after January 2020, the month that marks the explosion of the COVID pandemic in the Province of Padua.

## Materials and methods

### Study population

All patients with a diagnosis of MS with clinical onset between January 2020 and December 2020 were retrospectively enrolled in this cross-sectional study (pandMS) and matched for age at onset, gender, and disease duration, with a cohort of 20 patients with a diagnosis of MS before December 2019 (MS). A reference cohort of 20 individuals complaining tension headache, transient subjective sensory symptoms, and psychosomatic disorders, as well as unspecific white matter alterations, was also included in the study (12). Although no evidence of neurological or systemic disease was achieved in these subjects, they are defined as having other non-inflammatory neurological diseases (ONIND) rather than normal controls in this paper. Cerebrospinal fluid (CSF) and serum samples were obtained at the time of diagnosis. Therefore, no patient had ongoing disease-modifying medication or were treated with glucocorticoids in the last 28 days. The study was conducted in accordance with the Declaration of Helsinki and approved by the “Comitato Etico per la Sperimentazione Clinica dell’Azienda Ospedaliera di Padova” (Protocol Number AOP2108). All patients signed a written informed consent at study enrolment.

### CSF and serum routine analysis

Paired CSF and serum specimens were collected by non-traumatic lumbar puncture between 8:00 and 9:00 a.m., as previously described (13). Routine examination included the following: cell count and differentiation, CSF/serum IgG ratio (*Q*_IgG_), CSF/serum albumin ratio (*Q*_Alb_) to estimate the integrity of the blood–brain barrier (BBB), calculation of intrathecal IgG synthesis by means of quantitative formulas (IgG index) (14), IgG Reiber’s hyperbolic function for IgG intrathecal synthesis fraction (IgGIF), and local production (IgGLoc) and demonstration of IgGOB by isoelectric-focusing and specific IgG immunofixation. BBB damage was considered when *Q*_Alb_ was higher than the normal value for patient’s age (i.e., age/15 + 4), and expressed as a ratio (15). After cell centrifugation, both CSF and serum were stored at −80 °C until cytokine analysis.

### Cytokine determination

The CSF and serum levels of 45 cytokines were analyzed by means of ProcartaPlex Human Cytokine/Chemokine/Growth Factor Panel 1–45 plex, following the manufacturer’s instructions. The R&D Ella Automated Immunoassay System was applied to evaluate neurofilament light (NfL), BAFF, and CXCL13, in line with the manufacturer’s guidelines. The cytokines detected in more than 85% of samples were included in the analysis (13). For data analysis, when only CSF or serum concentration was available, that value was considered for further analysis. When both CSF and serum concentration were available, cytokine quotient (*Q*, cytokine CSF/cytokine serum) and then index (*Q*_cytokine_/*Q*_Alb_) were calculated. Specific analysis was performed when CSF concentrations were significantly higher than serum concentrations.

### Clinical and MRI evaluations

A subgroup of patients with MS underwent a brain and spinal cord MRI close to the lumbar puncture, as well as a neurological evaluation with the Expanded Disability Status Scale (EDSS) score. Images were acquired using a 3T scanner (Ingenia, Philips Medical Systems, Best, The Netherlands) with a 33-mT/m power gradient and a 32-channel head coil. No major hardware upgrades occurred during the study, and bimonthly quality assurance sessions assured measurement stability. The following images were acquired for each subject: (a) three-dimensional (3D) turbo field echo (TFE, 3D-T1): repetition time (RT) 7.8 ms; echo time (ET) 3.6 ms; 180 contiguous axial slices with the off-center positioned on zero with a thickness of 1.0 mm; flip angle = 8°; matrix size = 220 × 220; and FOV = 220 × 220 × 180 mm^3^. This sequence was acquired before and after gadolinium administration. (b) 3D-fluid attenuated inversion recovery (FLAIR): RT 4,800 ms; ET 310 ms; inversion time (IT) 1,650 ms; 365 contiguous axial slices with a thickness of 1.0 mm; matrix size = 256 × 256; and FOV = 256 × 256 × 182 mm^3^; (c) 3D-double inversion recovery (DIR): RT 13,000 ms; ET 10 ms; IT 3,400/325 ms; 40 contiguous axial slices; resolution 1 × 1 × 3 mm; FOV = 230 × 200 mm; and time 3.5 min. Spinal cord sequences evaluated both cervical and dorsal tract; the following sequences were acquired: (a) T2w: RT 4,117 ms; (b) ET 120 ms; thickness 3 mm; (c) T1w: RT 626 ms; ET 12 ms; thickness 3 mm; (d) STIR: RT 4,207 ms; ET 60 ms; IT 220 ms; thickness 3 mm. Two experienced observers, blinded to the patient’s identity, assessed all images. WM lesions were identified on FLAIR sequences, while cortical lesions were identified on DIR scans by two blinded evaluators (FDN and ADP) using published consensus recommendations. Tumefactive lesions were defined in the presence of a diameter > 2 cm.

### Statistical analysis

Comparisons of serum and CSF biomarker concentration between multiple groups were explored with analysis of variance (ANOVA) or Kruskal–Wallis test as appropriate, with Tukey’s or Dunn’s correction, respectively. A normal distribution test (Kolmogorov–Smirnov test) was performed to guide the choice of parametric or non-parametric test. Spearman correlation analysis was performed to test the association between cytokines (serum vs. CSF concentration as well as *Q*_cytokine_ vs. *Q*_Alb_). Multivariate logistic regression was used to find relevant independent explanatory cytokines for patients with MS. Only factors significantly associated with the outcome at univariate analysis were included in a multivariate model with a stepwise procedure. A *p*-value lower than 0.05 was considered statistically significant. SPSS (IBM) was used for all analyses.

## Results

### Study population

A total of 54 patients with a diagnosis of MS presented their clinical onset between January and December 2024 and were eligible for the study. Of them, 36 patients had available CSF and serum samples for cytokine analysis and were finally included in the study (pandMS). In addition, CSF and serum samples from 20 age-, gender-, and disease duration-matched patients with relapsing/remitting MS with an onset before 2020 (MS) and 20 ONIND were included in the study. Demographic parameters of all patients are reported in **Table 1**.

**Table 1:** Demographic and standard CSF parameters.

Demographic variables	ONIND	MS	pandMS	*p*-value
Sex ratio (F/M)	3.0	4.0	3.0	0.9419 ^a^
Age at LP	40.19 ± 16.02	36.62 ± 12.57	34.13 ± 10.07	0.2621 ^b^
Disease duration at LP (m)	n.a.	4.61 ± 3.50	2.92 ± 3.69	0.1104 ^b^
EDSS at LP	n.a.	1.5	1.5	0.4989 ^b^
IgGOB (%)	0%	100%	94%	<0.0001 ^a^
IgG index	0.56 ± 0.29	0.90 ± 0.42	0.89 ± 0.41	<0.0001 ^b^
*Q*_Alb_	5.87 ± 2.26	5.15 ± 2.33	5.07 ± 2.34	0.3499 ^b^

Disease duration was calculated as the interval between clinical disease onset and lumbar puncture and was expressed in months.

LP, lumbar puncture; y, years; EDSS, Expanded Disability Status Scale; m, months; IgGOB, IgG oligoclonal bands; Q_Alb_, albumin quotient. ^a^Chi-square test; ^b^Kruskal–Wallis test.

CSF and serum concentrations for each cytokine are reported in **Table 2**. Three molecules were detectable only in serum (S-EGF, S-PGFF-BB, and S-VEGF-D), while NfL concentration in CSF was defined in all patients (detectable concentrations: 100% in CSF, 73% in serum). When both CSF and serum values were available, cytokine quotient and then index was calculated for each cytokine. In the presence of CSF concentrations lower than serum, the cytokine index was considered (CXCL-13 index, CCL-3 index, CCL-4 index, CCL-11 index, CXCL-10 index, HGF-index, Kit-Ligand index, CXCL-12 index, and VEGF-A index). Three molecules had CSF concentration higher than serum 1 (CCL-2, CXCL-10, and IL-7), suggesting a physiological intrathecal synthesis. Finally, BAFF concentrations decreased in both MS groups.

**Table 2:** CSF and serum parameters.

Cytokines		ONIND	pandMS	MS	ANOVA	ONIND vs MS	ONIND vs pandMS	MS vs pandMS
BAFF	S	511.3 ± 122.2	468.7 ± 130.7	458.8 ± 132.9	0.556	>0.999	0.874	>0.999
	CSF	126.6 ± 53.6	72.0 ± 27.5	75.04 ± 25.37	<0.001	0.002	<0.001	>0.999
	Q	261.4 ± 140.0	161.3 ± 65.4	181.7 ± 100.2	0.002	0.024	0.002	>0.999
	Index	46.8 ± 20.8	37.1 ± 23.0	41.4 ± 23.0	0.187	>0.999	0.203	>0.999
CXCL-13	S	95.9 ± 85.7	68.8 ± 31.4	106.6 ± 99.3	0.210	0.975	0.414	0.293
	CSF	15.3 ± 15.3	42.8 ± 37.0	37.6 ± 32.6	0.003	0.030	0.002	>0.999
	Q	222.7 ± 353.9	803.3 ± 850.9	506.3 ± 439.1	<0.001	0.038	<0.001	>0.999
	Index	42.2 ± 59.1	167.0 ± 155.4	93.9 ± 99.6	<0.001	0.047	<0.001	0.504
EGF	S	190.5 ± 123.8	251.1 ± 170.4	148.0 ± 84.9	0.057	0.778	0.729	0.052
CCL-11	S	47.7 ± 39.3	65.8 ± 65.9	67.9 ± 60.4	0.346	0.521	0.717	>0.999
	CSF	0.9 ± 0.8	1.0 ± 1.0	2.3 ± 1.7	0.019	0.039	>0.999	0.033
	Q	19.1 ± 13.9	21.7 ± 31.4	34.8 ± 11.5	0.001	0.004	>0.999	0.001
	Index	3.2 ± 2.1	4.4 ± 5.2	7.8 ± 3.9	0.001	0.001	>0.999	0.001
HGF	S	551.4 ± 653.5	342.2 ± 253.0	511.6 ± 451.6	0.388	0.907	>0.999	0.539
	CSF	255.0 ± 96.4	765.9 ± 488.0	297.8 ± 143.2	<0.001	<0.001	0.822	<0.001
	Q	0.9 ± 0.6	1.2 ± 1.5	7.2 ± 16.8	<0.001	0.001	>0.999	0.001
	Index	0.2 ± 0.2	0.3 ± 0.4	1.5 ± 3.2	<0.001	<0.001	0.421	0.002
IL-7	S	4.4 ± 3.5	6.2 ± 5.5	2.5 ± 3.0	<0.001	0.052	0.574	<0.001
	CSF	109.5 ± 169.3	80.2 ± 92.1	97.4 ± 34.6	0.005	0.022	>0.999	0.006
	Q	41,891 ± 60,606	72,733 ± 81,372	20,460 ± 32,065	<0.001	0.027	0.918	<0.001
	Index	18,304 ± 58,446	4,602 ± 8,408	17,321 ± 22,334	0.004	0.026	0.951	0.001
CXCL-10	S	64.7 ± 73.7	69.1 ± 46.2	29.6 ± 27.9	<0.001	0.031	0.309	<0.001
	CSF	123.9 ± 88.2	445.3 ± 597.6	308.1 ± 150.6	<0.001	0.001	0.001	>0.999
	Q	2.8 ± 2.3	8.1 ± 11.7	15.2 ± 10.7	<0.001	<0.001	0.018	0.001
	Index	721.1 ± 676.2	1,846 ± 2,676	3,187 ± 2,428	<0.001	<0.001	0.048	0.002
CCL2	S	202.1 ± 236.3	152.1 ± 194.0	289.1 ± 224.0	0.003	>0.999	0.046	0.006
	CSF	1,208 ± 791.2	1,271 ± 530.7	1,436 ± 1,948	0.823	0.990	0.826	0.912
	Q	17.3 ± 21.1	8.37 ± 13.99	15.87 ± 13.99	<0.001	0.598	0.022	0.001
	Index	3,503 ± 4,846	1,982 ± 3,567	3,751 ± 2,964	0.002	0.4208	0.2144	0.002
CCL-3	S	15.1 ± 27.7	15.4 ± 24.7	8.4 ± 11.6	0.992	>0.999	>0.999	>0.999
	CSF	1.0 ± 0.7	2.7 ± 3.4	4.6 ± 3.4	0.001	0.001	0.044	0.024
	Q	515.4 ± 571.0	847.3 ± 1,708	1,600 ± 847.3	0.019	0.061	>0.999	0.029
	Index	63.3 ± 59.0	181.8 ± 403.9	379.3 ± 560.5	0.001	0.005	0.983	0.050
CCL-4	S	215.4 ± 297.9	167.7 ± 119.0	186.2 ± 92.30	0.249	0.287	>0.999	0.946
	CSF	19.6 ± 18.3	28.4 ± 35.6	88.5 ± 50.3	<0.001	<0.001	0.568	<0.001
	Q	154.0 ± 87.4	173.9 ± 108.9	477.6 ± 189.7	<0.001	<0.001	>0.999	<0.001
	Index	28.5 ± 20.8	109.1 ± 65.0	40.5 ± 30.4	<0.001	<0.001	0.543	<0.001
PDGF-BB	S	1,126 ± 665.7	1,655 ± 1,573	326.9 ± 189.1	<0.001	<0.001	>0.999	<0.001
SCF (KIT-ligand)	S	10.4 ± 8.0	11.1 ± 6.6	24.0 ± 23.4	0.001	0.004	0.981	0.003
	CSF	5.3 ± 3.1	4.1 ± 2.1	27.1 ± 18.89	<0.001	<0.001	0.890	<0.001
	Q	0.8 ± 0.9	0.6 ± 0.8	1.6 ± 1.1	<0.001	0.010	0.971	<0.001
	Index	156.3 ± 180.5	127.2 ± 132.1	409.2 ± 409.1	0.001	0.006	>0.999	0.001
CXCL-12	S	1,352 ± 851.1	1,399 ± 560.8	1,977 ± 846.3	0.019	0.029	>0.999	0.051
	CSF	1,483 ± 1,07	2,355 ± 1,645	5,516 ± 2,717	<0.001	<0.001	0.170	<0.001
	Q	1.0 ± 0.6	1.7 ± 0.9	2.7 ± 1.3	<0.001	<0.001	0.048	0.009
	Index	236.6 ± 209.9	390.8 ± 287.5	617.9 ± 518.6	<0.001	<0.001	0.043	0.101
VEGF-A	S	837.5 ± 725.6	975.3 ± 1,112	990.8 ± 944.2	>0.999	>0.999	>0.999	>0.999
	CSF	34.9 ± 26.3	30.6 ± 15.0	82.2 ± 57.9	<0.001	<0.001	>0.999	<0.001
	Q	81.4 ± 109.0	56.8 ± 47.9	143.7 ± 159.8	0.004	0.010	>0.999	0.008
	Index	21.0 ± 37.4	14.0 ± 14.8	32.8 ± 48.9	0.020	0.038	>0.999	0.0366
VEGF-D	S	42.3 ± 29.4	59.3 ± 62.1	36.9 ± 33.5	0.449	0.973	0.546	0.593
NfL	CSF	1,127 ± 1,869	1,94 ± 1,539	2,759 ± 4,44	0.004	0.023	0.005	>0.999

Concentrations are expressed in pg/mL as mean and standard deviation. Differences between multiple groups were explored with ANOVA or Kruskal–Wallis test as appropriate, with Tukey’s or Dunn’s correction respectively. S, serum; CSF, cerebrospinal fluid; Q, quotient; Index, cytokine Index.

### CCL-2 physiological intrathecal synthesis is influenced by serum CCL-2 only in pandMS

**Figure 1:**
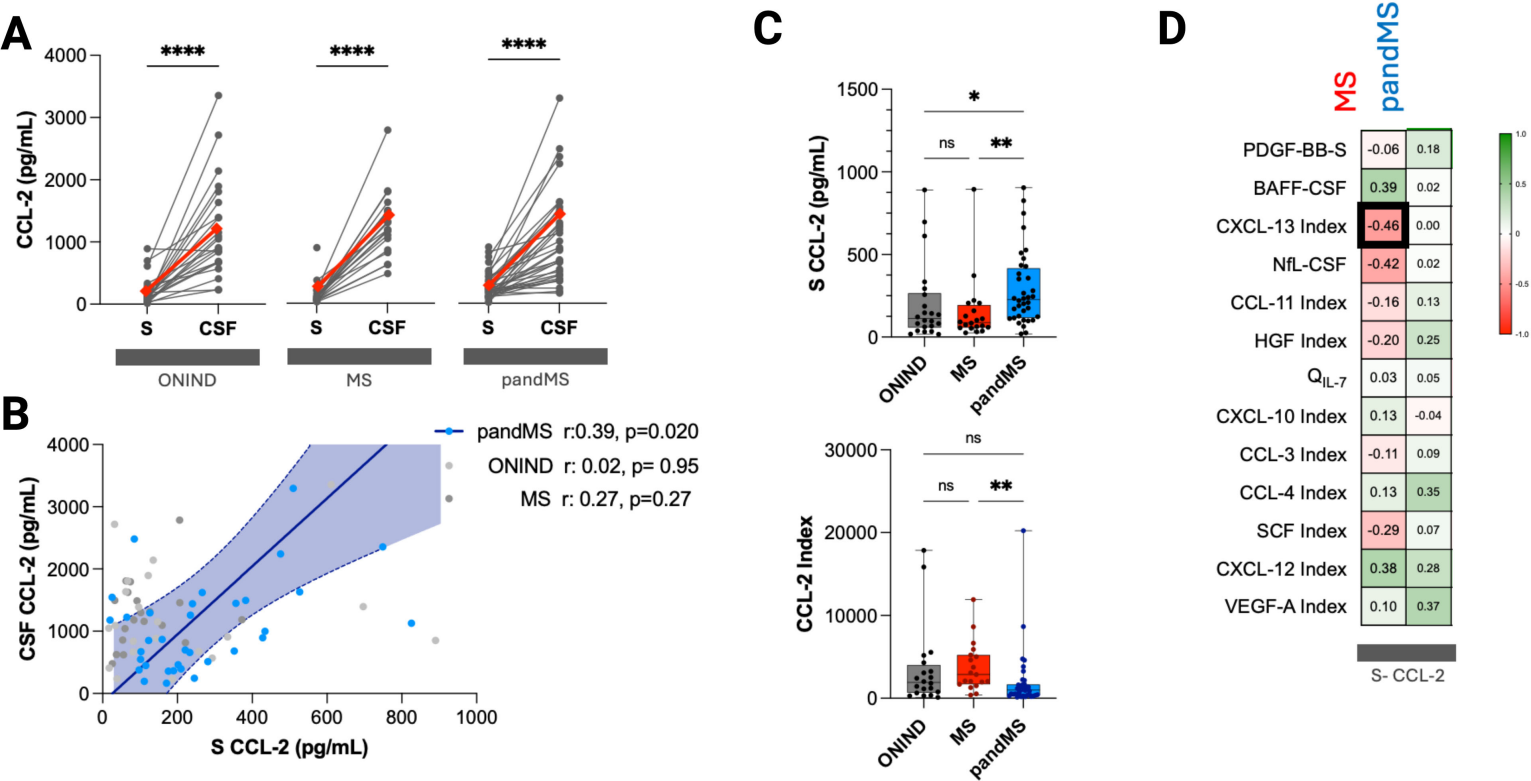
Peripheral CCL2 influences intrathecal levels specifically in patients with pandMS. **(A)** Paired comparison of CCL-2 levels in serum (S) and cerebrospinal fluid (CSF) for individual patients in ONIND, MS, and pandMS groups. Each line represents one patient, and red diamonds indicate median values. Significant differences between S and CSF are indicated (****p < 0.0001). **(B)** Correlation between serum and CSF CCL-2 levels in ONIND (gray), MS (dark gray), and pandMS (blue) patients. Linear regression lines with 95% confidence intervals are shown. Significant correlation was observed only in patients with pandMS (*r* = 0.39, *p* = 0.020). **(C)** Box plots showing serum CCL-2 levels (top) and CSF/serum CCL-2 index (bottom) across the three groups. Horizontal lines indicate median values, boxes represent interquartile ranges, and whiskers show minimum and maximum values. Statistical significance is indicated as *p < 0.05, **p < 0.01; ns = not significant. **(D)** Heatmap showing correlation coefficients between serum CCL-2 levels and various CSF biomarkers or indices in MS (red) and pandMS (blue) groups. Positive correlations are shown in green and negative correlations in red, with the intensity proportional to the correlation coefficient (*r*). Significant correlation is highlighted with thickened cell borders. Created in BioRender. Puthenparampil, (M) (2026) https://BioRender.com/yqf36l4.

CCL2 concentrations were consistently higher in CSF than in serum across all three groups (*p* < 0.001; **Figure 1A**). In ONIND and MS, serum and CSF concentrations did not correlate, supporting a predominantly intrathecal origin of CCL2. In contrast, pandMS displayed a significant association between serum and CSF CCL2 (**Figure 1B**), indicating a relevant contribution of serum CCL2 to intrathecal levels specifically in this group.

Serum CCL2 concentrations were significantly higher in pandMS compared with ONIND (*p* = 0.046) and MS (*p* = 0.006) (**Figure 1C**). Accordingly, these elevated serum levels resulted in reduced *Q*_CCL2_ and CCL2 index values in pandMS. Based on these findings, serum CCL2 was included in subsequent analyses (**Figure 1C**).

Finally, whereas MS showed a modest inverse correlation between serum CCL2 and the CXCL13 index (*r* = –0.46, *p* = 0.040), no associations between serum CCL2 and any other cytokine were observed in pandMS (**Figure 1D**).

### IL-7 concentrations show different pattern in MS and pandMS

**Figure 2:**
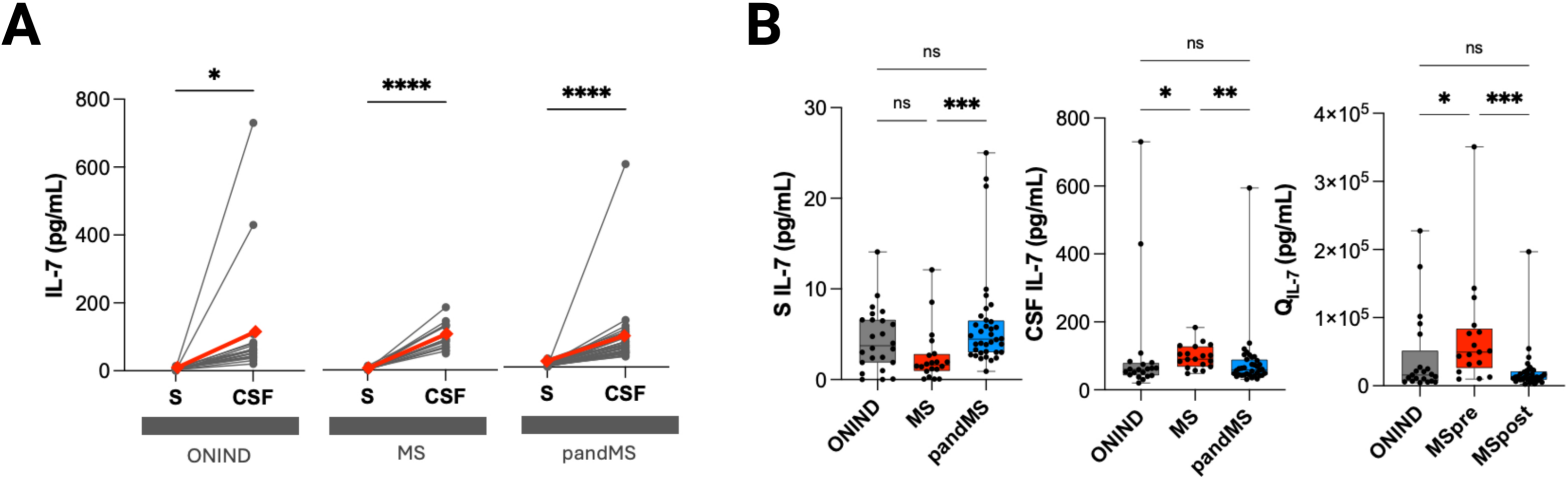
IL-7 levels in serum and CSF across patients with ONIND, MS, and pandMS. **(A)** Paired comparison of IL-7 concentrations in serum (S) and cerebrospinal fluid (CSF) for individual patients in ONIND, MS, and pandMS groups. Each line represents a single patient, and red diamonds indicate median values. Significant differences between S and CSF are indicated (*p < 0.05; ****p < 0.0001). **(B)** Box plots showing IL-7 levels in serum (S IL-7), CSF (CSF IL-7), and CSF/serum ratio (Q_IL-7) across the three groups. Horizontal lines represent median values, boxes indicate interquartile ranges, and whiskers show the minimum and maximum values. Statistical significance is indicated as *p < 0.05, **p < 0.01, ***p < 0.001; ns = not significant. Created in BioRender. Puthenparampil, (M) (2026) https://BioRender.com/4h0vb3y.

IL-7 concentrations were higher in CSF than in serum across all three groups (**Figure 2A**). Serum IL-7 levels were significantly increased in pandMS compared with MS (*p* < 0.001) (**Figure 2B**), whereas no correlation between serum and CSF IL-7 was observed in any group.

Conversely, CSF IL-7 concentrations were higher in MS than in both ONIND and pandMS. Given that serum IL-7 was specifically relevant in pandMS, whereas CSF IL-7 was more informative in MS, the IL-7 quotient was included in the subsequent analyses.

### The intrathecal synthesis of CXCL-10 is increased in both MS groups

**Figure 3:**
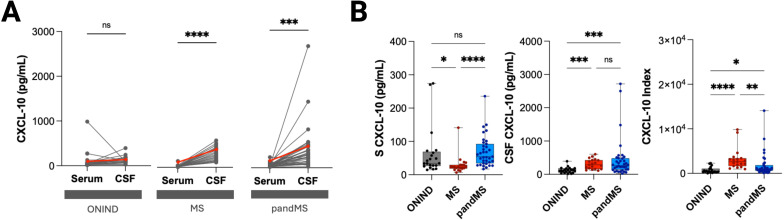
Marked intrathecal CXCL-10 synthesis characterizes multiple sclerosis. **(A)** Paired comparison of CXCL-10 levels in serum (S) and cerebrospinal fluid (CSF) for individual patients with non-inflammatory neurological disease (ONIND), multiple sclerosis (MS), and MS with pial inflammation (pandMS). Each line represents one patient, and red diamonds indicate median values. Significant differences between serum and CSF levels are indicated (**p < 0.001, *p* < 0.0001; ns = not significant). **(B)** Box plots showing serum CXCL-10 levels (left), CSF CXCL-10 levels (middle), and CSF/serum CXCL-10 index (right) across ONIND, MS, and pandMS groups. Horizontal lines indicate median values, boxes represent interquartile ranges, and whiskers show minimum and maximum values. Statistical significance is indicated as *p* < 0.05, *p* < 0.01, *p* < 0.001, *p* < 0.0001; ns = not significant. Created in BioRender. Puthenparampil, (M) (2026) https://BioRender.com/k8iy4oh.

CSF CXCL10 concentrations were significantly higher in both MS groups, whereas no difference was detected in ONIND (**Figure 3A**). In line with this, CSF CXCL10 levels exceeded serum levels in both MS and pandMS, but not in ONIND (**Figure 3B**). Across all groups, serum and CSF concentrations did not correlate (ONIND: *r* = 0.13, *p* = 0.59; MS: *r* = 0.16, *p* = 0.51; pandMS: *r* = 0.23, *p* = 0.18), indicating the absence of a serum-derived contribution.

These findings support the presence of pathological intrathecal CXCL10 synthesis in the MS groups, which was further evaluated using the CXCL10 index.

### Cytokine signatures differ between MS and pandMS

**Figure 4:**
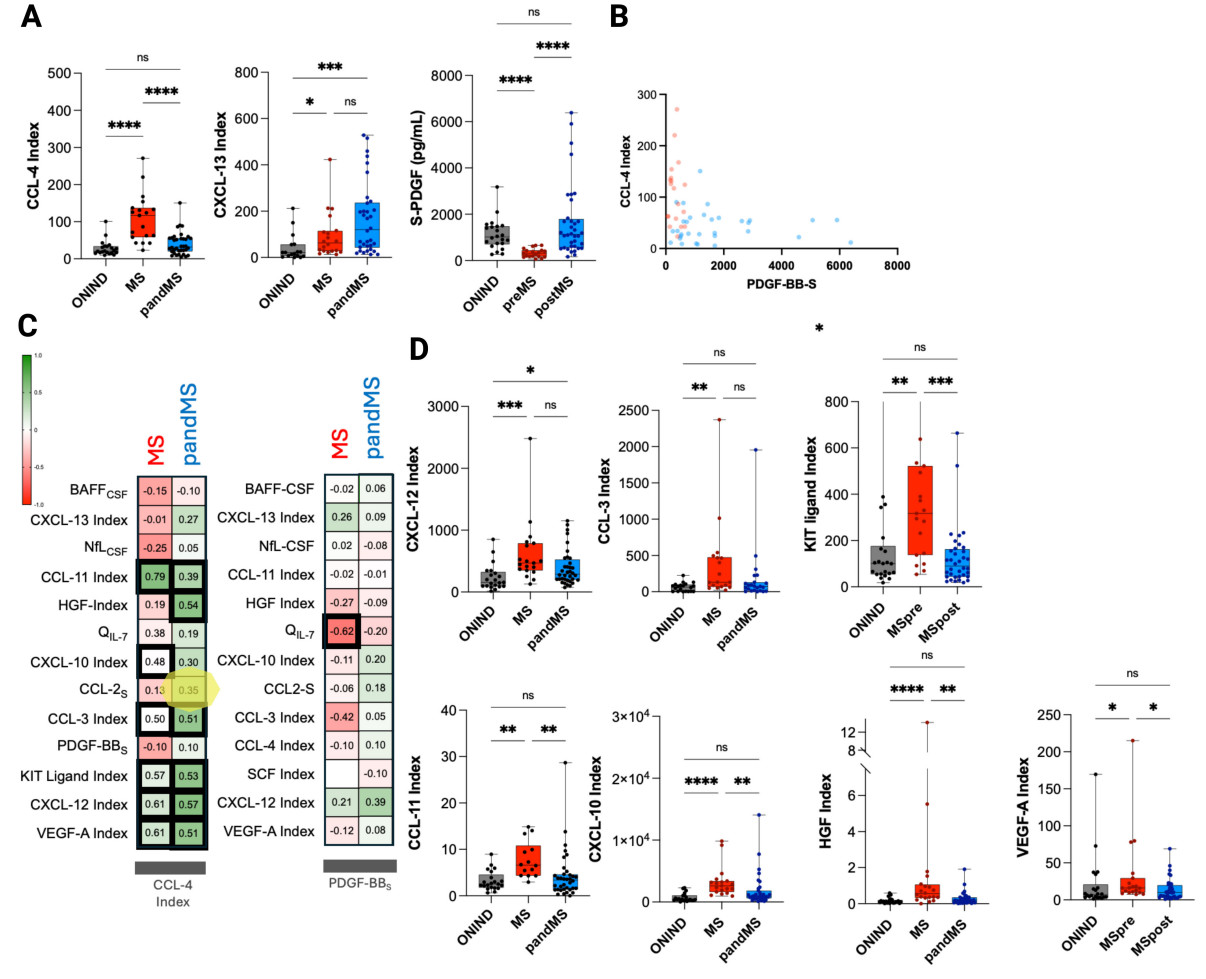
Cerebrospinal fluid (CSF) inflammatory, chemokine, and growth factor signatures in multiple sclerosis (MS) and their relationship with CCL-4 and PDGF-BB. **(A)** Box plots showing CSF CCL-4 index, CXCL-13 index, and soluble PDGF-BB (s-PDGF, pg/mL) levels across non-inflammatory neurological disease controls (ONIND), patients with multiple sclerosis (MS), and patients with MS with pial inflammation (pandMS), or across pre-MS and post-MS phases, as indicated. Horizontal lines indicate median values, boxes represent interquartile ranges, and whiskers show minimum and maximum values. Statistical significance between groups is indicated (*p* < 0.05, *p* < 0.01, *p* < 0.001, *p* < 0.0001; ns = not significant). **(B)** Correlation between CSF CCL-4 index and CSF PDGF-BB levels in patients with ONIND (gray), MS (red), and pandMS (blue). Individual data points are shown. **(C)** Heatmap showing Spearman correlation coefficients between CSF CCL-4 index (left) or CSF PDGF-BB levels (right) and selected CSF biomarkers or indices in MS (red) and pandMS (blue) groups. Positive correlations are shown in green and negative correlations are shown in red, with color intensity proportional to the correlation coefficient (*r*). Significant correlation is highlighted with thickened cell borders. **(D)** Box plots showing CSF levels or indices of CXCL-12, CCL-3, KIT ligand, CCL-11, CXCL-10, HGF, and VEGF-A across ONIND, MS, and MS subgroups, as indicated. Data are displayed as median with interquartile range and minimum–maximum values. Statistical significance is indicated as *p* < 0.05, *p* < 0.01, *p* < 0.001, *p* < 0.0001; ns = not significant. Created in BioRender. Puthenparampil, (M) (2026) https://BioRender.com/k9b9dy6.

To determine whether pandMS could be distinguished from MS, a logistic regression analysis was performed. The model demonstrated that the two groups could be separated (*r*² = 0.65) based on the CXCL13 index (*β* = 0.10, *p* < 0.001), as well as on the CCL4 index (*β* = 0.10, *p* = 0.010) and serum PDGF-BB levels (*β* = –0.01, *p* < 0.0005) (**Figures 4A, B**).

Correlation analysis (**Figure 4C**) revealed that in both MS groups, the CCL4 index correlated with the CCL3, CCL11, Kit-Ligand, CXCL12, and VEGF-A indices. However, these cytokines were significantly increased only in MS when compared with ONIND and pandMS (**Figure 4D**).

### Clinical and radiological parameters

Clinical parameters did not differ between the two groups (**Table 1**). A subgroup of 51 patients (18 MS and 33 pandMS) underwent brain and spinal cord MRI close to the time of lumbar puncture; among them, 48 patients (94.1%) also completed spinal cord imaging. The number and distribution of white matter lesions did not differ between groups (**Table 3**). Although the overall count of cortical lesions (CLs) was comparable (1.94 ± 3.32 vs. 0.76 ± 1.25, *p* = 0.06), CLs were more frequently detected in MS (66.7%) than in pandMS (36.4%; *p* = 0.0463).

**Table 3:** MRI parameters in MS and post MS.

MRI variables	MS (18 patients)	pandMS (33 patients)	p-value
Cortical Lesions (n)	1.94 ± 3.32	0.76 ± 1.25	0.06111^a^
Cortical Lesions (%)	66.7%%	36.4%	0.0463 ^b^
Total White matter lesions (n)	18.50 ± 14.57	13.15 ± 10.04	0.2134 ^a^
Total White matter lesions (%)	100%	100%	n.a.
Subcortical (n)	6.72 ± 7.60	3.54 ± 4.38	0.0900 ^a^
Subcortical (%)	88.9%	75.8%	0.4619 ^b^
Periventricular (n)	5.50 ± 5.08	5.30 ± 4.46	0.8869 ^a^
Periventricular (%)	94.4%	90.9%	>0.9999 ^b^
Infratentorial (n)	2.17 ± 2.85	1.88 ± 2.25	0.8592 ^a^
Infratentorial (%)	55.6%	66.7%	0.5474 ^b^
Optic Nerve (n)	0.44 ± 0.71	0.18 ± 0.39	0.2021 ^a^
Optic Nerve (%)	33.3%	18.2%	0.3036 ^b^
Spinal Cord (n)	1.72 ± 1.78	1.58 ± 2.15	0.5337 ^a^
Spinal Cord (%)	72.2%	66.7%	0.7776 ^b^
Cervical (n)	0.59 ± 0.87	0.97 ± 1.28	0.3383 ^a^
Cervical (%)	38.9%	54.5%	0.3823 ^b^
Dorsal (n)	1.24 ± 1.60	0.76 ± 1.38	0.3460 ^a^
Dorsal (%)	50.0%	45.5%	0.7776 ^b^
Gadolinium-anhancing Lesions (n)	0.50 ± 1.25	0.69 ± 0.97	0.1442 ^a^
Gadolinium-anhancing Lesions (%)	22.2%	48.5%	0.0802 ^b^
Tumefactive Lesions (n)	0.11 ± 0.32	0.03 ± 0.17	0.5436 ^a^
Tumefactive Lesions (%)	11.1%	3.0%	0.2816 ^b^

Number are expressed as mean ± standard deviation. a: Kruskal-Wallis test; b: Chi square test.

In the MS group, CSF NfL levels significantly correlated with total white matter lesion count (*r* = 0.53, *p* = 0.025), as well as with the presence of periventricular (*r* = 0.48, *p* = 0.045), juxtacortical–cortical (*r* = 0.59, *p* = 0.010), brainstem (*r* = 0.68, *p* = 0.002), and gadolinium-enhancing lesions (*r* = 0.54, *p* = 0.020). In contrast, in pandMS, serum CCL2 correlated with white matter lesion count (*r* = 0.28, *p* = 0.041) and with periventricular (*r* = 0.36, *p* = 0.037) and infratentorial lesions (*r* = 0.32, *p* = 0.039). No associations with EDSS were observed in either group.

## Discussion

Padua and its surrounding province were among the first Italian areas severely affected by the SARS-CoV-2 pandemic, beginning in February 2020. A SARS-CoV-2 vaccine only became available at the end of 2020. The first nationwide lockdown lasted from March to May 2020. Between June and December, before the second lockdown was implemented, a rapid and progressive rise in the number of swab-positive asymptomatic or pauci-symptomatic individuals, as well as clinically overt COVID-19 cases, was recorded. In the municipality of Vò—near Padua and known as the location of the first Italian COVID-19-related deaths—a strict lockdown was promptly instituted, and nasopharyngeal swabs were performed in approximately 80% of the population, revealing an infection prevalence of 2.6% (16). Notably, 42.5% of the SARS-CoV-2-positive individuals were asymptomatic. In a nearby area, a seroprevalence of 4.6% was reported (17). However, the rapid decline of serological titers and the large proportion of asymptomatic infections markedly hindered accurate estimation of the true infection prevalence. Beyond the direct impact of viral spread, the pandemic profoundly—albeit transiently—altered population behaviors and environmental exposures. These modifications involved several known MS-related environmental risk factors. For this reason, cytokine alterations observed in MS cases emerging in 2020 cannot be directly attributed to SARS-CoV-2 exposure alone (18). In addition to potential viral contact, the effects of lockdown measures must be considered. Social distancing, mask use, and sanitation procedures reduced interpersonal interactions and decreased levels of airborne and water pollutants (19, 20). Conversely, while particulate matter concentrations declined, body mass index (BMI) changed in nearly 70% of individuals during the lockdown (21). Altered physiological functions (e.g., sleep disruption and gastrointestinal irregularity) and widespread weight gain were interpreted as indicators of population-level psychological distress (22).

Within this environmental framework, we investigated cytokine expression in patients with MS who experienced their first clinical event between January and December 2020 (pandMS) and compared their profiles with those of patients whose MS onset occurred during the pre-pandemic period (2016–2019). Two patients developed severe symptomatic COVID-19, and four (12.1%) had detectable anti-SARS-CoV-2 antibodies. Interestingly, two patients who had symptomatic COVID-19 months before sampling lacked measurable antibody levels, further emphasizing the underestimation of SARS-CoV-2 infection prevalence.

Comprehensive CSF and serum analyses revealed two partially overlapping but distinct cytokine signatures in MS and pandMS. A small subset of cytokines—CXCL-13 and BAFF—was increased in both groups. These B-cell-related cytokines have well-established roles in MS pathogenesis (12, 13, 23). CXCL-13 mediates B-cell recruitment, whereas BAFF supports B-cell survival. We confirmed that CSF BAFF concentrations decrease at MS onset, a finding associated with CSF-infiltrating lymphocytes (particularly B cells) and intrathecal IgG synthesis in early disease phases (12, 23–25). In contrast, higher CSF BAFF levels have been associated with more advanced disease, likely reflecting the evolution of MS pathology (26, 27). Multiple regression analyses indicated that CXCL-13 may help discriminate pandMS from MS, likely due to the particularly elevated CSF CXCL-13 concentrations in pandMS, suggesting enhanced lymphocyte recruitment into the CNS.

A second cytokine cluster (including CCL-4, CCL-3, CCL-11, CXCL-10, CXCL-12, Kit-Ligand, and VEGF-A) showed significant intercorrelation in both groups but increased only in MS. This cluster includes CCL-3, whose CSF elevation has been linked to clinical and radiological inflammatory activity during RRMS follow-up, a finding reinforced here using a different analytical method and both CSF and serum matrices (13). CXCL1-0, produced by astrocytes (28) and reactive microglia (29), is a chemoattractant for macrophages, monocytes, and activated T and NK cells expressing CXCR3 (30–33). CXCL10–CXCR3 signaling enhances microglial activation in the cuprizone model of EAE (34), supporting CXCL-10 as a marker of astrocyte–microglia activation, potentially accompanied by CCL4 (35). *In vitro*, microglia release CCL-4 following LPS stimulation (36). A pro-inflammatory intrathecal role for CCL-4 is further supported by evidence from Alzheimer’s disease, where astrocyte-derived CCL-4 promotes microglial migration (36, 37), and from ischemic stroke, in which CCL-4 contributes to BBB disruption and CNS inflammation (38). In the central nervous system, CCL-11 (eotaxin-1) is primarily produced by activated astrocytes and microglia. Under inflammatory conditions, additional resident cells—including choroid plexus epithelial cells and pericytes—can contribute to local CCL-11 synthesis. Circulating CCL-11 is also capable of crossing the BBB and entering the CNS (39). In MS, this chemokine has been associated with mechanisms underlying progressive disease, likely reflecting compartmentalized intrathecal inflammation (40). Additional evidence supporting an intracerebral cytokine/chemokine network includes recent demonstrations of neuronal production of Kit tyrosine kinase and expression of its receptor KitR on microglia, astrocytes, oligodendrocytes, and neurons (41). VEGF-A, also included in this cytokine cluster, increases BBB permeability in EAE (42), and its receptors become upregulated on microglia following CNS trauma, implicating VEGF-A in CNS immune surveillance (35). Collectively, these data highlight the central role of astrocyte–microglia interactions in orchestrating CNS inflammation in MS (43).

CXCL-12 has also been implicated in MS immunopathogenesis (44). It supports plasma-cell maintenance through CXCR4 signaling (45) and promotes astrocyte secretion of pro-inflammatory cytokines (TNF-α, IL-1β, and CCL-5), contributing to inflammation and tissue injury (46). In the healthy mature CNS, CXCL12 modulates neurotransmission, neurotoxicity, and glial interactions (47). These findings reinforce the relevance of B-cell recruitment mechanisms in MS and suggest an even more prominent role in pandMS.

Unlike MS, pandMS displayed specific increases in peripheral IL-7, PDGF-BB, and CCL-2. Serum CCL-2 concentrations also correlated with CSF CCL-2, indicating either facilitated CNS entry or enhanced intrathecal production. The association between CCL-2 serum levels and white matter lesion load in pandMS further supports this interpretation. The prominence of peripheral cytokines in pandMS suggests systemic immune phenomena contributing to autoimmune activation, differentiating this phenotype from MS, which featured stronger astrocyte–microglia-driven intracerebral inflammation.

Although several behavioral and environmental factors associated with MS immunopathology changed—positively or negatively—during the SARS-CoV-2 pandemic, no direct causal link between viral exposure and pandMS onset can be established. Nevertheless, the temporal evolution of selected cytokines warrants attention, as it may reflect parallel modifications in the environmental risk-factor network and their contribution to MS clinical expression (2). This interpretation aligns with growing evidence supporting a significant influence of environmental factors on MS risk across several European regions, including the Province of Padua (48–50).

The main limitation of this study is the relatively small sample size, driven by the availability of SARS-CoV-2 vaccination from December 2020 onward and by the difficulty in clearly identifying individuals who developed MS after documented viral exposure.

In conclusion, we show that while certain cytokines previously associated with MS remain temporally stable, others exhibit variation across the pandemic period, likely reflecting concurrent changes in environmental exposures. These findings further underscore the relevance of environmental factors in MS immunopathogenesis.

## Data Availability

The raw data supporting the conclusions of this article will be made available by the authors, without undue reservation.

## References

[1] Waubant E Lucas R Mowry E Graves J Olsson T Alfredsson L . Environmental and genetic risk factors for MS: an integrated review. Ann Clin Transl Neurol. (2019), 6(9)acn3.50862. doi: 10.1002/acn3.50862, PMID: 31392849 PMC6764632

[2] Puthenparampil M Perini P Bergamaschi R Capobianco M Filippi M Gallo P . Multiple sclerosis epidemiological trends in Italy highlight the environmental risk factors. J Neurol. (2022) 269:1817–24. doi: 10.1007/S00415-021-10782-5, PMID: 34580756 PMC8940874

[3] Lucas RM Ponsonby AL Dear K Valery PC Pender MP Taylor BV . Sun exposure and vitamin D are independent risk factors for CNS demyelination. Neurology. (2011) 76:540–8. doi: 10.1212/WNL.0b013e31820af93d, PMID: 21300969

[4] Salzer J Hallmans G Nyström M Stenlund H Wadell G Sundström P . Smoking as a risk factor for multiple sclerosis. Multiple Sclerosis J. (2013) 19:1022–7. doi: 10.1177/1352458512470862, PMID: 23257617

[5] Bjornevik K Münz C Cohen JI Ascherio A . Epstein–Barr virus as a leading cause of multiple sclerosis: mechanisms and implications. Nat Rev Neurol. (2023) 19:160–71. doi: 10.1038/s41582-023-00775-5, PMID: 36759741

[6] Soldan SS Lieberman PM . Epstein–Barr virus and multiple sclerosis. Nat Rev Microbiol. (2022) 21:51–64. doi: 10.1038/s41579-022-00770-5, PMID: 35931816 PMC9362539

[7] Bjornevik K Cortese M Healy BC Kuhle J Mina MJ Leng Y . Longitudinal analysis reveals high prevalence of Epstein-Barr virus associated with multiple sclerosis. Science. (2022) 375:296–301. doi: 10.1126/SCIENCE.ABJ8222, PMID: 35025605

[8] Thomas OG Bronge M Tengvall K Akpinar B Nilsson OB Holmgren E . Cross-reactive EBNA1 immunity targets alpha-crystallin B and is associated with multiple sclerosis. Sci Adv. (2023) 9. doi: 10.1126/SCIADV.ADG3032, PMID: 37196088 PMC10191428

[9] Lünemann JD Jelčić I Roberts S Lutterotti A Tackenberg B Martin R . EBNA1-specific T cells from patients with multiple sclerosis cross react with myelin antigens and co-produce IFN-gamma and IL-2. J Exp Med. (2008) 205:1763–73. doi: 10.1084/JEM.20072397, PMID: 18663124 PMC2525578

[10] Marti Z Ruder J Thomas OG Bronge M de la Parra Soto L Grönlund H . Enhanced and cross-reactive *in vitro* memory B cell response against Epstein-Barr virus nuclear antigen 1 in multiple sclerosis. Front Immunol. (2024) 15:1334720. doi: 10.3389/FIMMU.2024.1334720, PMID: 39257578 PMC11385009

[11] Watanabe R Wege H Ter Meulen V . Adoptive transfer of EAE-like lesions from rats with coronavirus-induced demyelinating encephalomyelitis. Nature. (1983) 305:150–3. doi: 10.1038/305150A0, PMID: 6310411 PMC7094959

[12] Puthenparampil M Federle L Miante S Zito A Toffanin E Ruggero S . BAFF Index and CXCL13 levels in the cerebrospinal fluid associate respectively with intrathecal IgG synthesis and cortical atrophy in multiple sclerosis at clinical onset. J Neuroinflamm. (2017) 14:11. doi: 10.1186/s12974-016-0785-2, PMID: 28095856 PMC5240243

[13] Puthenparampil M Stropparo E Zywicki S Bovis F Cazzola C Federle L . Wide cytokine analysis in cerebrospinal fluid at diagnosis identified CCL-3 as a possible prognostic factor for multiple sclerosis. Front Immunol. (2020) 11:174. doi: 10.3389/FIMMU.2020.00174, PMID: 32194540 PMC7066207

[14] Link H Tibbling G . Principles of albumin and IgG analyses in neurological disorders. III. Evaluation of IgG synthesis within the central nervous system in multiple sclerosis. Scand J Clin Lab Invest. (1977) 37:397–401. doi: 10.1080/00365517709091498, PMID: 337461

[15] Puthenparampil M Tomas-Ojer P Hornemann T Lutterotti A Jelcic I Ziegler M . Altered CSF albumin quotient links peripheral inflammation and brain damage in MS. Neurol - Neuroimmunology Neuroinflamm. (2021) 8. doi: 10.1212/NXI.0000000000000951, PMID: 33649179 PMC7963437

[16] Lavezzo E Franchin E Ciavarella C Cuomo-Dannenburg G Barzon L Del Vecchio C . Suppression of a SARS-CoV-2 outbreak in the Italian municipality of Vo’. Nature. (2020) 584:425–9. doi: 10.1038/s41586-020-2488-1, PMID: 32604404 PMC7618354

[17] Plebani M Padoan A Fedeli U Schievano E Vecchiato E Lippi G . SARS-CoV-2 serosurvey in health care workers of the Veneto Region. Clin Chem Lab Med. (2020) 58:2107–11. doi: 10.1515/CCLM-2020-1236/MACHINEREADABLECITATION/RIS 32845861

[18] MacDougall M El-Hajj Sleiman J Beauchemin P Rangachari M . SARS-coV-2 and multiple sclerosis: potential for disease exacerbation. Front Immunol. (2022) 13:871276/BIBTEX. doi: 10.3389/FIMMU.2022.871276/BIBTEX 35572514 PMC9102605

[19] Barouki R Kogevinas M Audouze K Belesova K Bergman A Birnbaum L . The COVID-19 pandemic and global environmental change: Emerging research needs. Environ Int. (2021) 146:106272. doi: 10.1016/J.ENVINT.2020.106272, PMID: 33238229 PMC7674147

[20] Rume T Islam SMDU . Environmental effects of COVID-19 pandemic and potential strategies of sustainability. Heliyon. (2020) 6. doi: 10.1016/J.HELIYON.2020.E04965, PMID: 32964165 PMC7498239

[21] Bolesławska I Jagielski P Błaszczyk-Bębenek E Jagielska A Przysławski J . Lifestyle Changes during the SARS-CoV-2 Pandemic as Predictors of BMI Changes among Men and Women in Poland. Nutrients. (2023) 15. doi: 10.3390/NU15112427/S1 PMC10254752 37299391

[22] Pellegrino A Rosselli M Orlandi M Boddi M Stefani L Toncelli L . PHYSICAL ACTIVITY AND LIFESTYLE CHANGES IN ITALY DURING THE COVID-19 LOCKDOWN: GENDER DIFFERENCES. J Hypertens. (2022) 40:e169. doi: 10.1097/01.HJH.0000837096.34518.73, PMID: 33975735

[23] Puthenparampil M Miante S Federle L Zanetta C Toffanin E Ruggero S . BAFF is decreased in the cerebrospinal fluid of multiple sclerosis at clinical onset. J Neuroimmunol. (2016) 297:63–7. doi: 10.1016/J.JNEUROIM.2016.05.013, PMID: 27397077

[24] Kowarik MC Cepok S Sellner J Grummel V Weber MS Korn T . CXCL13 is the major determinant for B cell recruitment to the CSF during neuroinflammation. J Neuroinflamm. (2012) 9:624. doi: 10.1186/1742-2094-9-93, PMID: 22591862 PMC3418196

[25] Ragheb S Li Y Simon K Vanhaerents S Galimberti D De RM . Multiple sclerosis: BAFF and CXCL13 in cerebrospinal fluid. Multiple Sclerosis J. (2011) 17(7):819–29. doi: 10.1177/1352458511398887, PMID: 21372118

[26] Paul A Comabella M Gandhi R . Biomarkers in multiple sclerosis. Cold Spring Harb Perspect Med. (2019) 9:a029058. doi: 10.1101/cshperspect.a029058, PMID: 29500303 PMC6396336

[27] Farina G Magliozzi R Pitteri M Reynolds R Rossi S Gajofatto A . Increased cortical lesion load and intrathecal inflammation is associated with oligoclonal bands in multiple sclerosis patients: a combined CSF and MRI study. J Neuroinflamm. (2017) 14. doi: 10.1186/S12974-017-0812-Y, PMID: 28222766 PMC5319028

[28] Salmaggi A Ciusani E De Rossi M Gelati M Dufour A Corsini E . Expression and modulation of IFN-gamma-inducible chemokines (IP-10, Mig, and I-TAC) in human brain endothelium and astrocytes: possible relevance for the immune invasion of the central nervous system and the pathogenesis of multiple sclerosis. J Interferon Cytokine Res. (2002) 22:631–40. doi: 10.1089/10799900260100114, PMID: 12162873

[29] Cheeran MC-J Hu S Sheng WS Peterson PK Lokensgard JR . CXCL10 production from cytomegalovirus-stimulated microglia is regulated by both human and viral interleukin-10. J Virol. (2003) 77:4502. doi: 10.1128/JVI.77.8.4502-4515.2003, PMID: 12663757 PMC152158

[30] Sorensen TL Trebst C Kivisäkk P Klaege KL Majmudar A Ravid R . Multiple sclerosis: A study of CXCL10 and CXCR3 co-localization in the inflamed central nervous system. J Neuroimmunol. (2002) 127:59–68. doi: 10.1016/S0165-5728(02)00097-8, PMID: 12044976

[31] Sørensen TL Sellebjerg F Jensen CV Strieter RM Ransohoff RM . Chemokines CXCL10 and CCL2: differential involvement in intrathecal inflammation in multiple sclerosis. Eur J Neurol. (2001) 8:665–72. doi: 10.1046/J.1468-1331.2001.00327.X, PMID: 11784351

[32] Simpson JE Newcombe J Cuzner ML Woodroofe MN . Expression of the interferon-gamma-inducible chemokines IP-10 and Mig and their receptor, CXCR3, in multiple sclerosis lesions. Neuropathol Appl Neurobiol. (2000) 26:133–42. doi: 10.1046/J.1365-2990.2000.026002133.X, PMID: 10840276

[33] Scarpini E Galimberti D Baron P Clerici R Ronzoni M Conti G . IP-10 and MCP-1 levels in CSF and serum from multiple sclerosis patients with different clinical subtypes of the disease. J Neurol Sci. (2002) 195:41–6. doi: 10.1016/S0022-510X(01)00680-3, PMID: 11867072

[34] Clarner T Janssen K Nellessen L Stangel M Skripuletz T Krauspe B . CXCL10 triggers early microglial activation in the cuprizone model. J Immunol. (2015) 194:3400–13. doi: 10.4049/JIMMUNOL.1401459, PMID: 25725102

[35] Puthenparampil M Torresin T Franciotta S Marin A De Napoli F Mauceri VA . Hyper-reflecting foci in multiple sclerosis retina associate with macrophage/microglia-derived cytokines in cerebrospinal fluid. Front Immunol. (2022) 13:852183/FULL. doi: 10.3389/FIMMU.2022.852183/FULL 35664007 PMC9160385

[36] Peterson PK Hu S Salak-Johnson J Molitor TW Chao CC . Differential production of and migratory response to β Chemokines by human microglia and astrocytes. J Infect Dis. (1997) 175:478–81. doi: 10.1093/INFDIS/175.2.478, PMID: 9203678

[37] Xia M Qin S Wu L Mackay CR Hyman BT . Immunohistochemical study of the β-chemokine receptors CCR3 and CCR5 and their ligands in normal and alzheimer’s disease brains. Am J Pathol. (1998) 153:31. doi: 10.1016/S0002-9440(10)65542-3, PMID: 9665462 PMC1852933

[38] Estevao C Bowers CE Luo D Sarker M Hoeh AE Frudd K . CCL4 induces inflammatory signalling and barrier disruption in the neurovascular endothelium. Brain Behav Immun Health. (2021) 18:100370. doi: 10.1016/J.BBIH.2021.100370, PMID: 34755124 PMC8560974

[39] Nazarinia D Behzadifard M Gholampour J Karimi R Gholampour M . Eotaxin-1 (CCL11) in neuroinflammatory disorders and possible role in COVID-19 neurologic complications. Acta Neurol Belg. (2022) 122:865–9. doi: 10.1007/s13760-022-01984-3, PMID: 35690992 PMC9188656

[40] Huang J Khademi M Fugger L Lindhe Ö Novakova L Axelsson M . Inflammation-related plasma and CSF biomarkers for multiple sclerosis. Proc Natl Acad Sci U.S.A. (2020) 117:12952–60. doi: 10.1073/pnas.1912839117, PMID: 32457139 PMC7293699

[41] Al-Muhsen SZ Shablovsky G Olivenstein R Mazer B Hamid Q . The expression of stem cell factor and c-kit receptor in human asthmatic airways. Clin Exp Allergy. (2004) 34:911–6. doi: 10.1111/j.1365-2222.2004.01975.x, PMID: 15196279

[42] Argaw AT Asp L Zhang J Navrazhina K Pham T Mariani JN . Astrocyte-derived VEGF-A drives blood-brain barrier disruption in CNS inflammatory disease. J Clin Invest. (2012) 122:2454–68. doi: 10.1172/JCI60842, PMID: 22653056 PMC3386814

[43] Beckner ME . Factors promoting tumor angiogenesis. Cancer Invest. (1999) 17:594–623. doi: 10.3109/07357909909032845, PMID: 10592767

[44] Krumbholz M Theil D Cepok S Hemmer B Kivisäkk P Ransohoff RM . Chemokines in multiple sclerosis: CXCL12 and CXCL13 up-regulation is differentially linked to CNS immune cell recruitment. Brain. (2006) 129:200–11. doi: 10.1093/brain/awh680, PMID: 16280350

[45] Hargreaves DC Hyman PL Lu TT Ngo VN Bidgol A Suzuki G . A coordinated change in chemokine responsiveness guides plasma cell movements. J Exp Med. (2001) 194:45–56. doi: 10.1084/JEM.194.1.45, PMID: 11435471 PMC2193440

[46] Han Y He T Huang DR Pardo CA Ransohoff RM . TNF-alpha mediates SDF-1 alpha-induced NF-kappa B activation and cytotoxic effects in primary astrocytes. J Clin Invest. (2001) 108:425–35. doi: 10.1172/JCI12629, PMID: 11489936 PMC209361

[47] Li M Ransohoff RM . Multiple roles of chemokine CXCL12 in the central nervous system: A migration from immunology to neurobiology. Prog Neurobiol. (2008) 84:116. doi: 10.1016/J.PNEUROBIO.2007.11.003, PMID: 18177992 PMC2324067

[48] Tateo F Grassivaro F Ermani M Puthenparampil M Gallo P . PM2.5 levels strongly associate with multiple sclerosis prevalence in the Province of Padua, Veneto Region, North-East Italy. Multiple Sclerosis J. (2018), 135245851880327. doi: 10.1177/1352458518803273, PMID: 30270719

[49] Puthenparampil M Seppi D Rinaldi F Federle L Calabrese M Perini P . (MuSEV) MSEVSG. Increased incidence of multiple sclerosis in the Veneto region, Italy. Mult Scler. (2013) 19:601–4. doi: 10.1177/1352458512461970, PMID: 23599184

[50] Grassivaro F Puthenparampil M Pengo M Saiani M Venturini M Stropparo E . Multiple sclerosis incidence and prevalence trends in the province of padua, northeast Italy, 1965-2018. Neuroepidemiology. (2019) 52:41–6. doi: 10.1159/000493857, PMID: 30476909

